# circRNA circ‐CCND1 promotes the proliferation of laryngeal squamous cell carcinoma through elevating CCND1 expression via interacting with HuR and miR‐646

**DOI:** 10.1111/jcmm.14925

**Published:** 2020-01-17

**Authors:** Yanzi Zang, Jing Li, Baoluo Wan, Yong Tai

**Affiliations:** ^1^ Department of Otolaryngology People's Hospital of Henan Province Zhengzhou China

**Keywords:** CCND1/cyclin D1, Circular RNA, mRNA stability, prognosis, proliferation

## Abstract

Cyclin D1 (CCND1) is a well‐known proliferation promoter that accelerates G1/S transition in cancer. However, the underlying mechanism by which CCND1 is regulated is still largely unknown. In this study, we identified a novel circular RNA (circRNA) derived from CCND1 (circ‐CCND1, hsa_circ_0023303) as a key regulator for CCND1. circ‐CCND1 was found to be markedly up‐regulated in laryngeal squamous cell carcinoma (LSCC) and closely associated with aggressive clinical features and adverse prognosis. Depletion of circ‐CCND1 significantly inhibited LSCC cell proliferation in vitro and retarded tumour growth in vivo*.* Regarding the mechanism, circ‐CCND1 physically bound to human antigen R (HuR) protein to enhance CCND1 mRNA stability; on the other hand, circ‐CCND1 could act as an effective sponge for miR‐646 to alleviate the repression of miR‐646 on CCND1 mRNA. As a result, circ‐CCND1 post‐transcriptionally elevated CCND1 expression via coordinated avoidance of CCND1 mRNA decay, thereby promoting LSCC tumorigenesis. Taken together, our findings uncover the essential proliferation‐promoting role of circ‐CCND1 through regulation of the stability of CCND1 mRNA in LSCC. Targeting circ‐CCND1 may be a promising treatment for LSCC patients.

**Abbreviations**
circRNA: circular RNA
HuR: human antigen R
LSCC: laryngeal squamous cell carcinoma

## 1 INTRODUCTION

Laryngeal squamous cell carcinoma (LSCC) is a common malignant tumour of head and neck.^1^ In recent decades, the number of patients diagnosed with LSCC has continued to substantially increase, which has become a worldwide public health problem.^2^ The 5‐year survival rate of LSCC patients is still dismal, especially in advanced patients.^3^ Therefore, a better understanding of the pathogenesis of LSCC will provide new diagnostic and prognostic indicators to tackle this thorny disease.

Cyclin D1 (CCND1), a highly conserved cyclin family protein, has been recognized as indispensable for the transition of cell cycle from G1 phase into S phase.^4^ CCND1 is able to form a ternary complex with cyclin‐dependent kinases CDK4 and CDK6 to phosphorylate Rb and release E2F transcription factors, thus facilitating G1/S transition.^5^ It has been well documented that CCND1 is a proto‐oncogene that is frequently overexpressed in various human cancers, including LSCC.^6^ Dysregulation of CCND1 can lead to uncontrolled cell proliferation and malignancy, which enables it to be a feasible and effective therapeutic target for cancer.^7^ Although CCND1 is closely linked to tumorigenesis and aggressiveness, the underlying mechanism regulating the function of CCND1 in LSCC still remains largely elusive.

As a special class of non‐coding RNA, circular RNA (circRNA), characterized by the covalently closed ring structure, is attracting considerable attention.^8^ circRNA is highly stable and conservative with regulatory potency.^9^ A large number of studies have shown that circRNA is involved in the development and progression of human diseases, especially in cancer.^10^ Emerging evidence suggests that circRNA can modulate its parental gene expression in a *cis‐* or *trans‐*acting manner. For instance, circ‐ITGA7 up‐regulated its linear isoform ITGA7 via the miR‐370‐3p/neurofibromin 1/Ras pathway in colorectal cancer.^11^ circ‐FLI1 was capable of directly interacting with TET1 and DNMT1 to coordinately increase FLI1 expression in breast cancer.^12^ circ‐PABPN1 was reported to inhibit the translation of PABPN1 by reducing the binding of HuR and PABPN1 mRNA.^13^ These studies imply that there is a crosstalk between circRNA and its host gene.

In the present study, we screened CCND1‐derived circRNAs and identified that circ‐CCND1 (hsa_circ_0023303) was highly expressed and could elevate CCND1 in LSCC. The potential mechanism by which circ‐CCND1 regulated its host gene was further elucidated.

## 2 MATERIALS AND METHODS

### 2.1 Ethical standards

This study was approved by the Ethics Committee of People's Hospital of Henan Province and complied with the principles of the Helsinki Declaration. Handwritten informed consent was provided by each enrolled patient. Besides, the animal study was carried out with approval of the Animal Care Committee of People's Hospital of Henan Province.

### 2.2 LSCC tissues and cell lines

A total of 101 pairs of carcinoma and noncancerous tissues were collected from patients who were diagnosed as LSCC and underwent surgical resection. Among them, 25 matched tissues were used to determine the aberrantly expressed in LSCC. All surgically removed tissues were stored in liquid nitrogen before use. We routinely followed up patients every three months. The detailed clinicopathological features of patients are described in Table 1. LSCC cell lines (AMC‐HN‐8, Hep‐2, LSC‐1, TU212, TU177, TU686, SCC10A) and the normal NP‐69 cells and 293T cells were all purchased from Chinese Academy of Sciences (Shanghai, China). Cells were routinely grown as per the manufacturer's protocols, and mycoplasma examination was conducted every 3 months.

**Table 1:** Correlation between circ‐CCND1 expression and clinicopathological features of patients with LSCC

Parameters	All cases (n = 101)	circ‐CCND1 expression		*P* value
		Low (n = 51)	High (n = 50)	
Gender				
Male	54	28	26	.770
Female	47	23	24	
Age (years)				
≤60	52	25	27	.617
>60	49	26	23	
Smoking status				
No	44	23	21	.754
Yes	57	28	29	
T classification				
T1‐T2	63	43	20	.000
T3‐T4	38	8	30	
TNM stage				
I‐II	59	37	22	.004
III‐IV	42	14	28	
Tumour differentiation				
Well	32	23	9	.007
Moderate	46	21	25	
Poor	23	7	16	

### 2.3 Quantitative real‐time polymerase chain reaction (qRT‐PCR)

Total RNA was isolated with TRIzol reagent (Invitrogen), and the relative quantification of RNA expression was performed with SYBR Premix EX Taq (Takara) using the 2^−ΔΔCt^ method. Actin and U6 were used as internal reference for circRNA/mRNA and miRNA, respectively. The primer sequences used in this study are presented in Table 2.

**Table 2:** Primer sequences used in this study

Gene	Direction	Sequence (5′‐3′)
hsa_circ_0023303	Forward	TCCTCTCCAAAATGCCAGAG
	Reverse	ACTCTGCTGCTCGCTGCTAC
hsa_circ_0023304	Forward	GCGGAGGAGAACAAACAGAT
	Reverse	AGGAAGCGGTCCAGGTAGTT
hsa_circ_0023305	Forward	TTCCCAGCACCAACATGTAA
	Reverse	AGGAAGCGGTCCAGGTAGTT
CCND1	Forward	AACTACCTGGACCGCTTCCT
	Reverse	TCGGTGTAGATGCACAGCTT
CCND1‐5′‐UTR	Forward	CTGGAGCCTCCAGAGGGCTGT
	Reverse	GCGCTCCCTCGCGCTCTTC
CCND1‐CDS	Forward	ACGAAGGTCTGCGCGTGTT
	Reverse	CCGCTGGCCATGAACTACCT
CCND1‐3′‐UTR	Forward	GGAAAGCTTCATTCTCCTTGTTG
	Reverse	TCTTTTGCTTAAGTCAGAGATGGAA
CCND1‐Intron 1	Forward	CTTTGTTCAAGCAGCGAGTC
	Reverse	AAGGTCCTCCAAGCCGATA
CCND1‐Intron 2	Forward	CCCAGCTCCCTTGAGTCC
	Reverse	CGGTCCTGGATGTTGGAG
CCND1‐Intron 3	Forward	TTTGTCATCGGCCAGAAATA
	Reverse	GACCTTCAGAGCACAGACCA
Actin	Forward	GACCTGACTGACTACCTCATGAAGAT
	Reverse	GTCACACTTCATGATGGAGTTGAAGG
miR‐646	Forward	GAAGCAGCTGCCTCTGAGC
	Reverse	CAGAGCGCCAGCGAGGAGCC
U6	Forward	GCTTCGGCAGCACATATACTAAAAT
	Reverse	CGCTTCACGAATTTGCGTGTCAT

### 2.4 Cell transfection

All oligomers used in this study including siRNA targeting circ‐CCND1 junction site, miR‐646 mimics and inhibitors were commercially obtained from GenePharma Co., Ltd. To overexpress CCND1 and HuR, the full length of them was inserted into pCMVp‐NEO‐BAN expression vector (Biovector), respectively. The aforementioned oligomers and plasmids were individually or jointly transfected into Hep‐2 and TU212 cells using Lipofectamine 3000 (Invitrogen) as per the manufacturer's protocols.

### 2.5 Colony formation and cell cycle

For colony formation assay, 500 Hep‐2 and TU212 cells transfected with si‐NC or si‐circ‐CCND1 were plated into six‐well plates and routinely grown for 10 days. Then, the cells were washed, fixed with methanol and stained with crystal violet. For cell cycle analysis, the treated cells were fixed in 75% pre‐cooled ethanol and then incubated with propidium iodide solution, followed by calculation for the cell proportion in G0/G1, S and G2/M phases by a flow cytometer.

### 2.6 Xenograft model

A total of 5 × 10^6^ Hep‐2 cells were subcutaneously injected into nude mice (n = 8 in each group). After one week, the subcutaneous tumours were visible and then intratumorally injected with cholesterol‐coupled si‐NC or si‐circ‐CCND1 (GenePharma) three times weekly for two weeks. Two weeks after the last injection of cholesterol‐coupled oligomers, all mice were killed and the tumours were carefully dissected, followed by weighing and photography.

### 2.7 Western blot

Western blot was employed to detect the protein expression of CCND1 and HuR after corresponding treatment. The assay was performed according to the standard protocols. The primary antibodies used in this study were as follows: anti‐CCND1 (#2922, CST, 1:1000 dilution), anti‐HuR (#12582, CST, 1:2000 dilution) and anti‐β‐actin (#3700, CST, 1:5000 dilution). β‐Actin was used as loading control.

### 2.8 RNA pull‐down and RIP assays

For RNA pull‐down assay, the biotin‐labelled probes were designed and synthesized (GenePharma) and then incubated with the lysates of 2 × 10^7^ treated Hep‐2 and TU212 cells overnight at 37°C, followed by addition of C1 streptavidin magnetic beads (Invitrogen) for 2 hours at 25°C. The protein enriched by probe was subjected to Western blot analysis for HuR expression. RIP assay was conducted with 5 μg anti‐HuR antibody (#12582, CST) using the RIP^™^ RNA‐Binding Protein Immunoprecipitation Kit (Merck Millipore) in accordance with the manufacturer's instructions.

### 2.9 Luciferase reporter assay

The full‐length sequence of circ‐CCND1 or CCND1‐3′‐UTR with wild‐type or mutant miR‐646 binding site was cloned into psiCHECK2 luciferase reporter (Promega) and named as Luc‐circ‐CCND1‐WT (5′‐AGCUGCU‐3′), Luc‐circ‐CCND1‐Mut (5′‐UCGACGA‐3′), Luc‐CCND1‐3′‐UTR‐WT (5′‐AGCUGCU‐3′) and Luc‐CCND1‐3′‐UTR‐Mut (5′‐AGCUGCU‐3′), respectively. The above vectors were cotransfected with control or miR‐646 mimics into Hep‐2 and TU212 cells using Lipofectamine 3000 (Invitrogen). After 48 hours, the luciferase activity was detected by a dual‐luciferase reporter system (Promega).

### 2.10 Data statistics

The comparison between groups was carried out by chi‐square test or Student's *t* test. The survival curve was plotted by the Kaplan‐Meier method and calculated by log‐rank test (LSCC patients were divided into two cohorts according to the median circ‐CCND1 expression in LSCC tissues, and death was used as the event of interest). Spearman's correlation model was employed to evaluate the correlation between circ‐CCND1 and CCND1 mRNA expression in LSCC tissues. Data analysis and presentation were performed with SPSS v22.0 and GraphPad Prism v5.0 software, and *P* value less than 0.05 was considered statistically significant.

## 3 RESULTS

### 3.1 circ‐CCND1 (hsa_circ_0023303), a circRNA derived from CCND1, is significantly up‐regulated in LSCC

**Figure 1:**
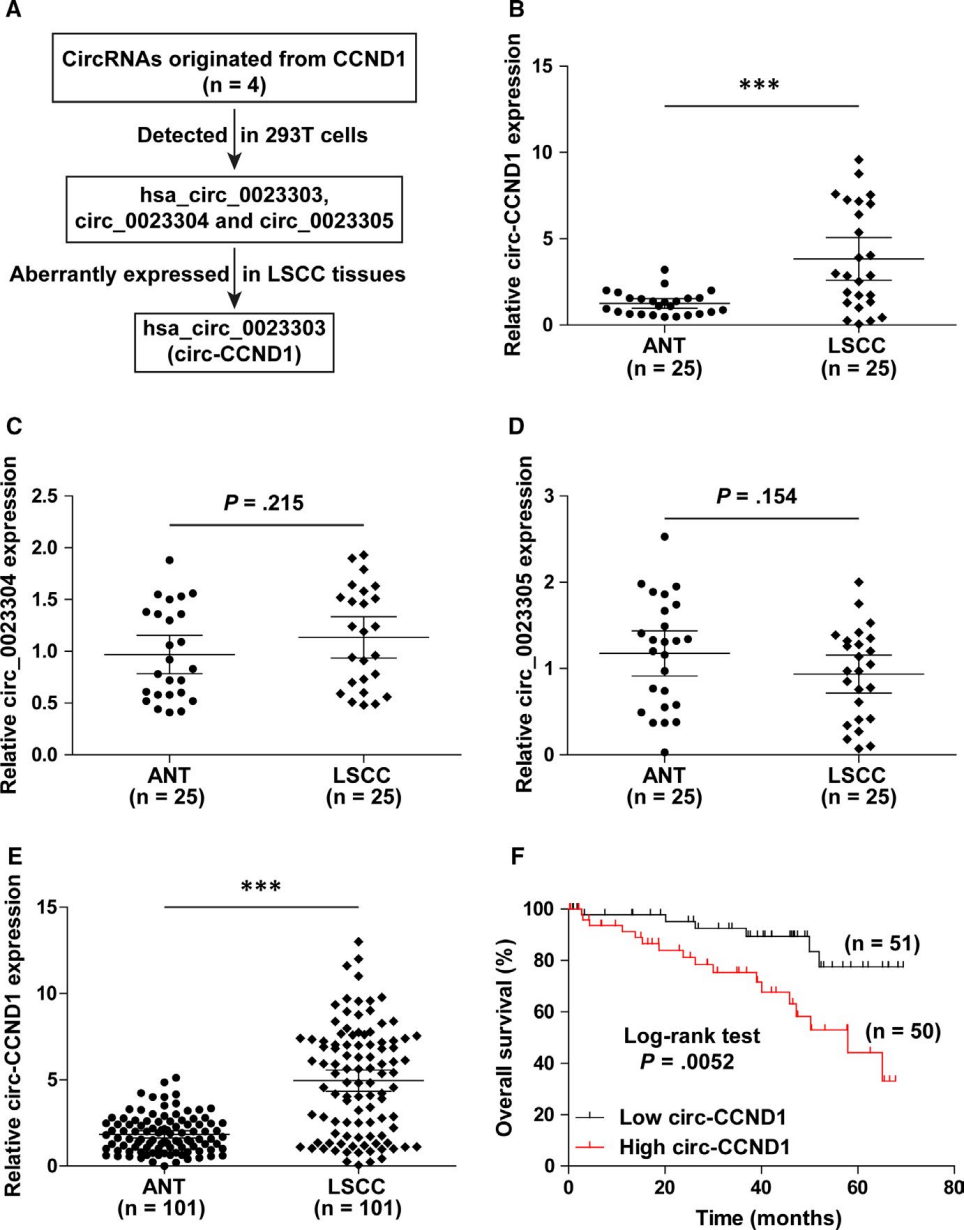
circ‐CCND1 is frequently overexpressed in LSCC and predicts poor prognosis. A, The flow chart showing the identification of circ‐CCND1 in LSCC. B‐D, qRT‐PCR analysis for the expression of circ‐CCND1, hsa_circ_0023304 and hsa_circ_0023305 in 25 paired LSCC and adjacent normal tissues (ANT). E, qRT‐PCR analysis for circ‐CCND1 expression in 101 pairs of LSCC and normal tissues. F, Kaplan‐Meier plotter showing the survival curve of LSCC patients with high and low circ‐CCND1 expression. *** *P* < .001

By searching for the circBase database (http://www.circbase.org/), we found that a total of four circRNAs were generated from CCND1. Among them, three (circ‐CCND1, hsa_circ_0023304 and hsa_circ_0023305) could be detected in human 293T cells. We then tested the expression levels of these three circRNAs in 25 LSCC and adjacent normal tissues (Figure 1A). The qRT‐PCR results showed that only circ‐CCND1 (Figure 1B), but not hsa_circ_0023304 and hsa_circ_0023305 (Figure 1C,D), was significantly aberrantly expressed in LSCC when compared to normal tissues. To further confirm this finding, we additionally collected 76 matched LSCC and normal tissues (101 cases in total) to assess circ‐CCND1 expression, and the results showed that circ‐CCND1 was markedly up‐regulated in LSCC tissues in comparison with precancerous tissues (Figure 1E). Importantly, high circ‐CCND1 was positively correlated with larger tumour size (*P* < .001), poor differentiation (*P* = .007) and advanced TNM stage (*P* = .004; Table. 1). Moreover, Kaplan‐Meier plotter showed that LSCC patients with high circ‐CCND1 expression had shorter overall survival time than those with low circ‐CCND1 expression (Figure 1F). In all, these data suggest that circ‐CCND1 is a dysregulated circRNA in LSCC and may be used as a promising biomarker.

### 3.2 Depletion of circ‐CCND1 delays LSCC cell growth both in vivo and in vitro

**Figure 2:**
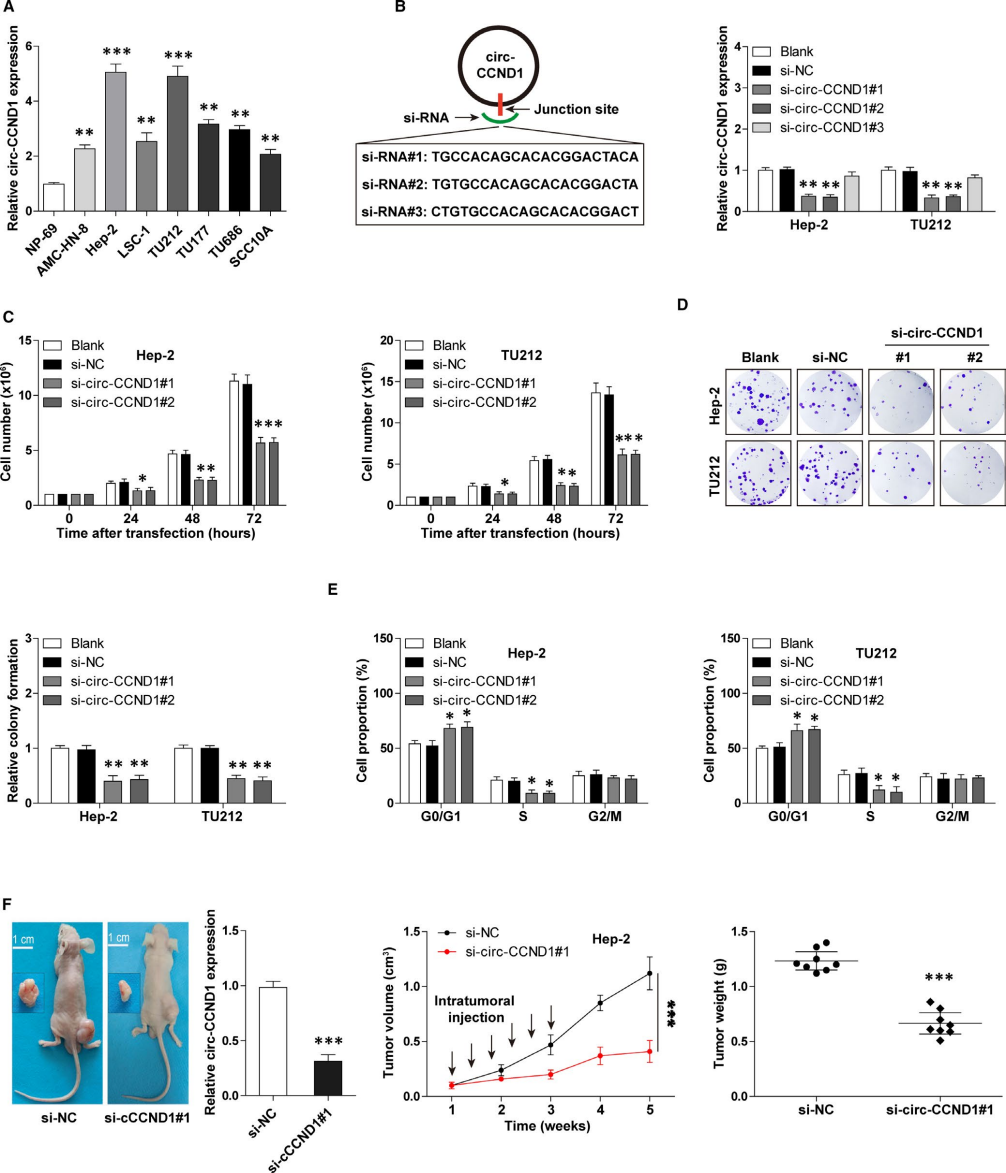
Silencing of circ‐CCND1 retards LSCC cell growth both in vitro and in vivo. A, qRT‐PCR analysis for circ‐CCND1 expression in LSCC cell lines. B, The diagram showing three designed siRNAs targeting the junction site of circ‐CCND1 (left). qRT‐PCR analysis verifying the knockdown efficiency of si‐circ‐CCND1#1 and #2 (right). C, The cell number counted at the indicated time after circ‐CCND1 depletion. D and E, The colony formation and cell cycle assays in circ‐CCND1‐depleted Hep‐2 and TU212 cells. F, The representative image showing the nude mice bearing tumours injected with cholesterol‐coupled control or si‐circ‐CCND1, the expression levels of circ‐CCND1 and the volume and weight of subcutaneous tumours in each group were analysed. * *P* < .05, ** *P* < .01, *** *P* < .001

To explore the function of circ‐CCND1, we first tested its expression in LSCC cell lines. Likewise, up‐regulated circ‐CCND1 expression was observed in seven LSCC cells as compared to normal NP‐69 cells, especially in Hep‐2 and TU212 cells (Figure 2A). We then designed three siRNAs targeting the junction sequence of circ‐CCND1 to knock down circ‐CCND1 (Figure 2B), and the results showed that si‐circ‐CCND1#1 and #2, but not si‐circ‐CCND1#3, could effectively reduce circ‐CCND1 expression by about 70% in both Hep‐2 and TU212 cells (Figure 2B). The decreased number of cells as well as clones was observed after circ‐CCND1 silencing (Figure 2C,D). In addition, cell cycle analysis displayed that depletion of circ‐CCND1 resulted in more cell arrest in G0/G1 phase (Figure 2E). Further, we established the xenograft model to explore the function of circ‐CCND1 in vivo. As expected, the volume and weight of subcutaneous tumours in the circ‐CCND1‐depleted group were less than those in the control group (n = 8 in each group) (Figure 2F). Taken together, these functional experiments reveal that circ‐CCND1 is a positive regulator for LSCC cell proliferation.

### 3.3 circ‐CCND1 elevates CCND1 via stabilization of CCND1 mRNA

**Figure 3:**
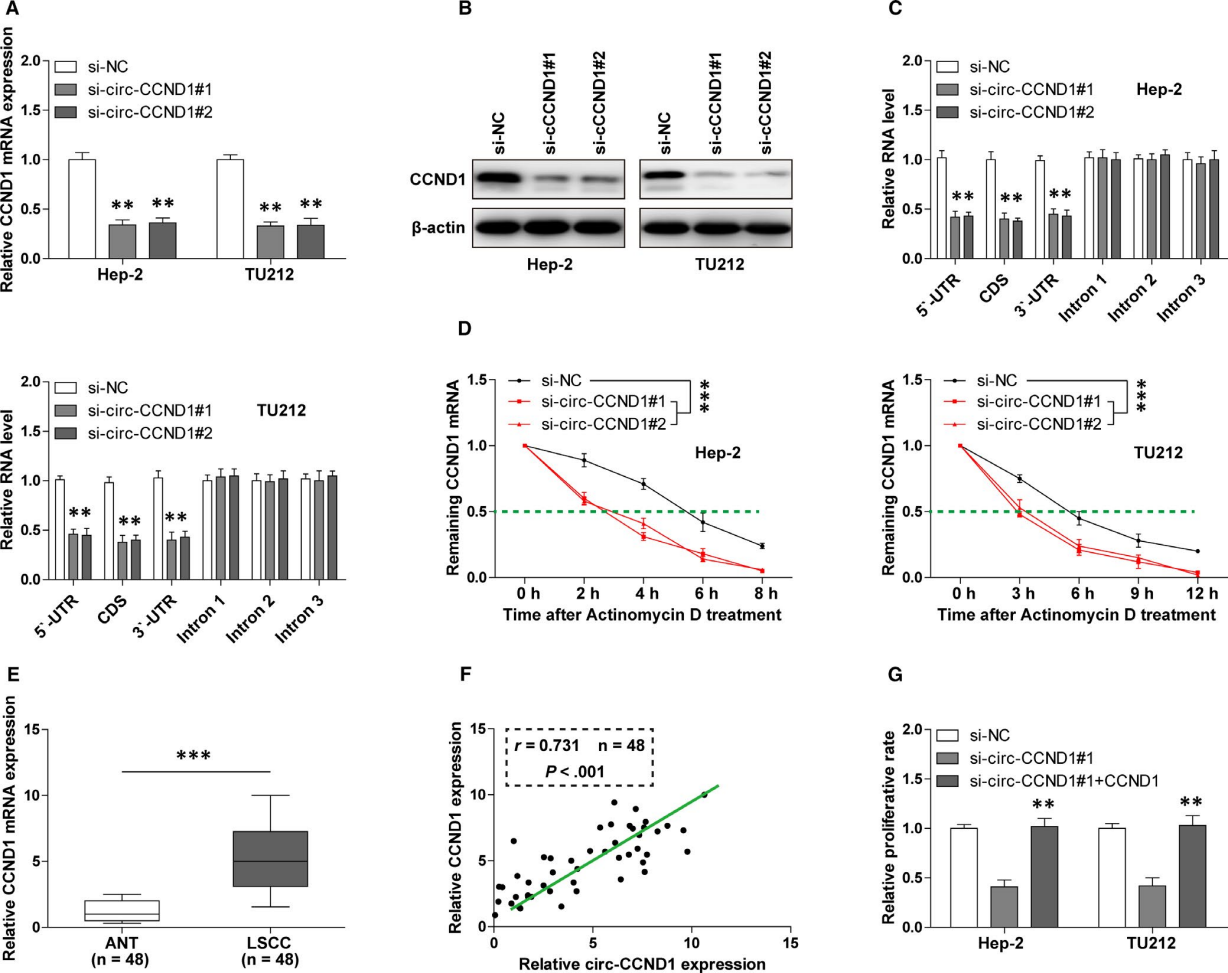
circ‐CCND1 regulates the stability of CCND1 mRNA. A, B, The mRNA expression and protein expression of CCND1 in control and circ‐CCND1‐silenced Hep‐2 and TU212 cells were detected by qRT‐PCR and Western blot assays, respectively. C, qRT‐PCR analysis for the different part of CCND1 mRNA in control and circ‐CCND1‐silenced Hep‐2 and TU212 cells. D, qRT‐PCR analysis for CCND1 mRNA expression in control and circ‐CCND1‐silenced Hep‐2 and TU212 cells at the indicated time after treatment with 1 mg/mL actinomycin D. E, qRT‐PCR analysis for CCND1 mRNA expression in 48 matched LSCC and normal tissues. F, The correlation between circ‐CCND1 and CCND1 mRNA expression in 48 LSCC tissues. G, The proliferative capabilities of circ‐CCND1‐silenced Hep‐2 and TU212 cells after transfection with CCND1 expression plasmid. ** *P* < .01, *** *P* < .001

Next, we tested whether CCND1 was modulated by circ‐CCND1. As shown in Figure 3A,B silencing of circ‐CCND1 dramatically decreased CCND1 mRNA as well as protein expression in Hep‐2 and TU212 cells. To decipher how circ‐CCND1 altered CCND1 mRNA expression, we designed a series of primers for different regions of CCND1 mRNA for qRT‐PCR analysis. The results showed that the level of mature CCND1 mRNA containing 5′‐UTR, CDS and 3′‐UTR was decreased after depletion of circ‐CCND1, whereas that of unspliced CCND1 pre‐mRNA containing introns remained unchanged (Figure 3C), suggesting that circ‐CCND1 regulates its host gene at the post‐transcriptional level. We then tested the stability of CCND1 mRNA via treating cells with actinomycin D to block de novo transcription, and the results showed that knockdown of circ‐CCND1 reduced the half‐life of CCND1 mRNA from about 5.5 hours to 3 hours (Figure 3D). Besides, we found that CCND1 mRNA expression was significantly up‐regulated in LSCC tissues (Figure 3E) and strongly positively correlated with circ‐CCND1 expression (Figure 3F). Moreover, the decreased proliferative abilities of Hep‐2 and TU212 cells caused by circ‐CCND1 depletion were completely rescued after exogenous expression of CCND1 (Figure 3G). Altogether, these results indicate that circ‐CCND1 is a stabilizer for CCND1 in LSCC.

### 3.4 circ‐CCND1 physically interacts with HuR to enhance the binding of HuR to CCND1 mRNA

**Figure 4:**
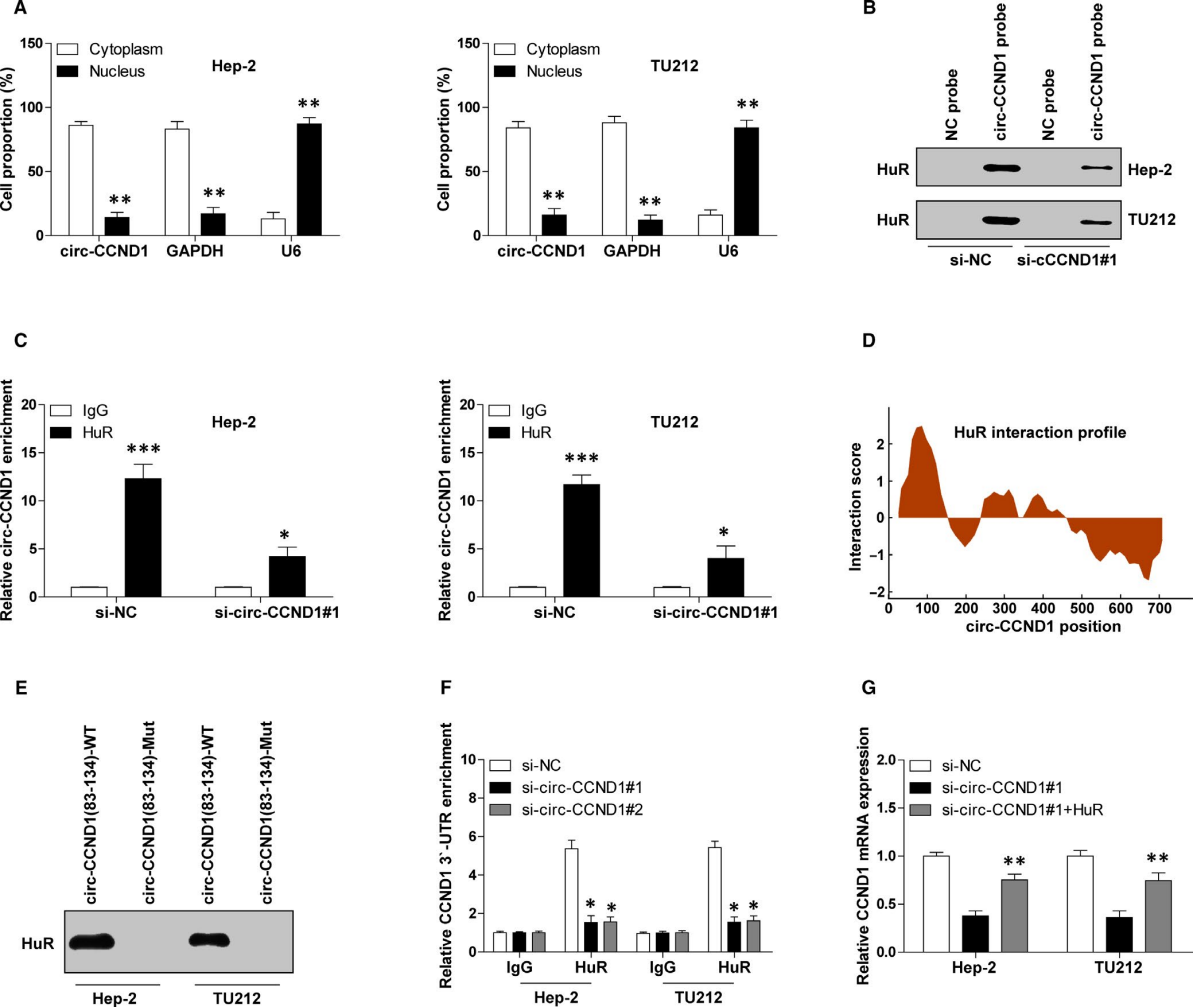
circ‐CCND1 interacts with HuR to increase CCND1 mRNA stability. A, qRT‐PCR analysis for circ‐CCND1 expression in the cytoplasmic and nuclear fractions. B, RNA pull‐down assay in control and circ‐CCND1‐silenced Hep‐2 and TU212 cells, followed by detection of HuR expression using Western blot assay. C, RIP assay in control and circ‐CCND1‐silenced Hep‐2 and TU212 cells, followed by qRT‐PCR analysis for circ‐CCND1 expression. D, The interaction profile between HuR and circ‐CCND1 predicted by online catRAPID algorithm. E, RNA pull‐down assay in Hep‐2 and TU212 cells with indicated wild‐type or mutated probe, followed by Western blot analysis for HuR protein expression. F, RIP assay in control and circ‐CCND1‐silenced Hep‐2 and TU212 cells with HuR or IgG antibody, followed by qRT‐PCR analysis for CCND1 3′‐UTR enrichment. G, qRT‐PCR analysis for CCND1 mRNA expression in control and circ‐CCND1‐silenced Hep‐2 and TU212 cells transfected with HuR expression vector. * *P* < .05, ** *P* < .01, *** *P* < .001

To determine how circ‐CCND1 increased CCND1 mRNA stability, we first detected the subcellular localization of circ‐CCND1. As shown in Figure 4A, circ‐CCND1 was predominately localized in the cytoplasm, which is consistent with its post‐transcriptional regulation of CCND1. By the online CircInteractome tool, we found that circ‐CCND1 can interact with some RNA‐binding proteins, in which human antigen R (HuR) attracted our attention mainly due to its function of RNA stabilization.^14^ RNA pull‐down results showed that a large amount of HuR protein was enriched by circ‐CCND1 probe, but not control probe, and it was diminished when circ‐CCND1 was depleted (Figure 4B). To further confirm this interaction, we performed RIP assay, and the results showed that more circ‐CCND1 was precipitated by HuR antibody in comparison with IgG negative control antibody in both Hep‐2 and TU212 cells (Figure 4C). Through online catRAPID algorithm, HuR was predicted to likely bind to the 83‐134 bp of circ‐CCND1 (Figure 4D). To test this prediction, we conducted RNA pull‐down assay and found that mutation of this region of circ‐CCND1 blocked the interaction between circ‐CCND1 and HuR (Figure 4E). More importantly, we found that depletion of circ‐CCND1 dramatically decreased the binding of HuR to CCND1 3′‐UTR (Figure 4F). In addition, ectopic expression of HuR could partially rescue the reduced CCND1 mRNA level caused by circ‐CCND1 knockdown (Figure 4G). Overall, these data suggest that HuR mediates the regulation of circ‐CCND1 on CCND1 mRNA stability.

### 3.5 circ‐CCND1 serves as a sponge for miR‐646

**Figure 5:**
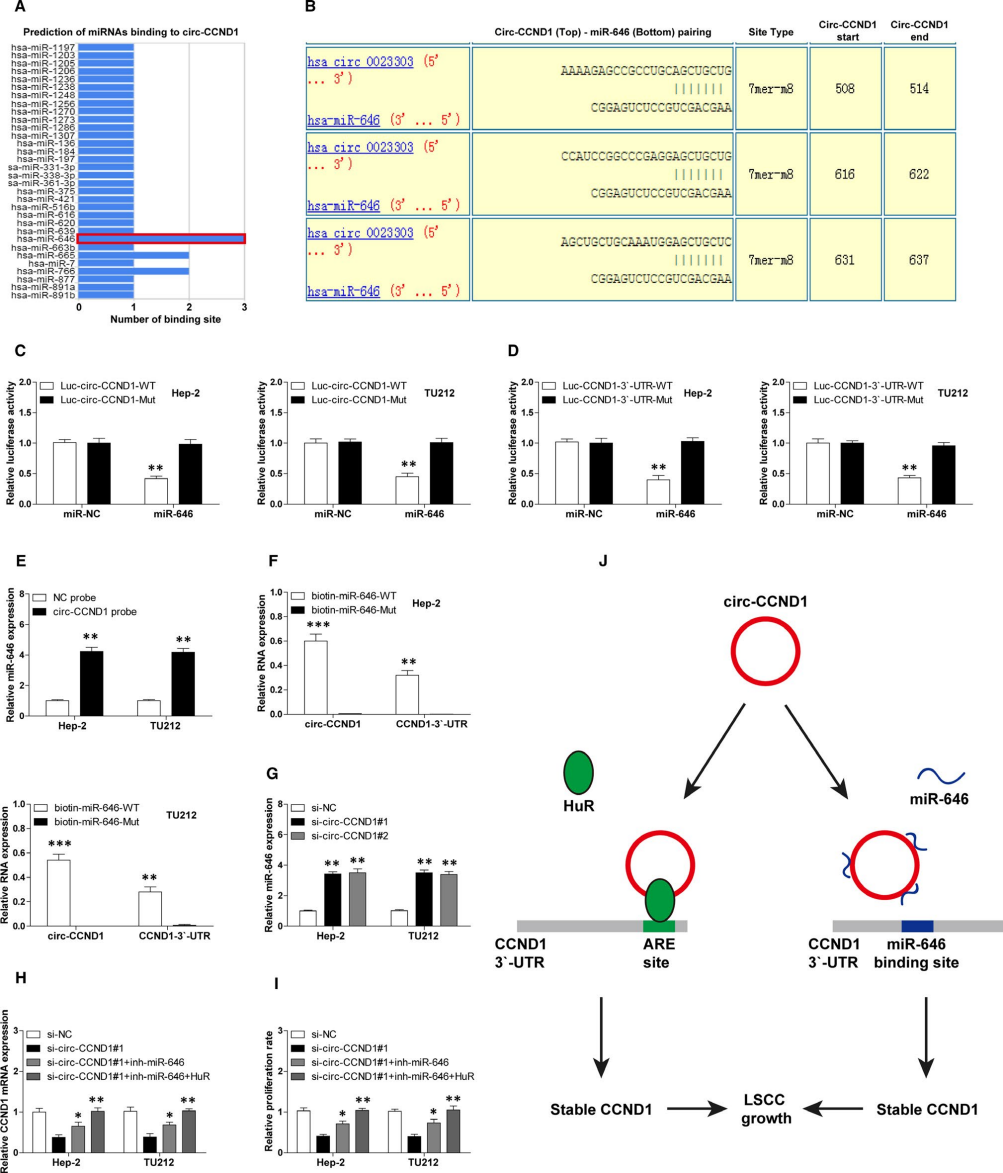
circ‐CCND1 functions as a sponge for miR‐646. A, The predicted miRNAs bound by circ‐CCND1 using the online CircInteractome tool. B, The specific binding site between circ‐CCND1 and miR‐646. C and D, The luciferase reporter assay in Hep‐2 and TU212 cells cotransfected with control or miR‐646 mimics and wild‐type or mutant circ‐CCND1/CCND1 luciferase vector. E. RNA pull‐down assay in Hep‐2 and TU212 cells using control or circ‐CCND1 probe, followed by qRT‐PCR analysis for miR‐646 expression. F, RNA pull‐down assay in Hep‐2 and TU212 cells transfected with wild‐type or mutant miR‐646 mimics, followed by qRT‐PCR analysis for the enrichment of circ‐CCND1 and CCND1 3′‐UTR. G, qRT‐PCR analysis for miR‐646 expression in control and circ‐CCND1‐silenced Hep‐2 and TU212 cells. H. qRT‐PCR analysis for CCND1 mRNA expression in circ‐CCND1‐silenced Hep‐2 and TU212 cells transfected with miR‐646 inhibitors or combined with HuR expression vector. I, The proliferative capabilities of circ‐CCND1‐silenced Hep‐2 and TU212 cells after transfection with miR‐646 inhibitors or combined with HuR expression vector. J, The schematic illustration of the proposed model depicting the crucial role of circ‐CCND1 in LSCC growth through regulating CCND1 mRNA stability via interaction with HuR and miR‐646. * *P* < .05, ** *P* < .01

As mentioned above, overexpression of HuR can only partially up‐regulate si‐circ‐CCND1‐induced decreased CCND1 mRNA expression, suggesting there are other mechanisms involved in the regulation of CCND1 stability by circ‐CCND1. Given that cytoplasmic circRNA is able to sponge miRNA and miRNA can reduce the mRNA stability via targeting the 3′‐UTR of mRNA, we thus inferred that miRNA might be involved in the regulation of circ‐CCND1 on CCND1 mRNA stability. By using the online CircInteractome tool, we found that several miRNAs had circ‐CCND1 binding sites, of which miR‐646 had the largest number of binding sites (Figure 5A,B). And CCND1 mRNA 3′‐UTR was also predicted to be bound by miR‐646 using the miRanda and TargetScan databases. We then performed luciferase reporter assay to evaluate the relationship between circ‐CCND1, miR‐646 and CCND1. The results showed that overexpression of miR‐646 dramatically reduced the luciferase activity of circ‐CCND1 or CCND1 vector with wild‐type miR‐646 binding site, whereas it had no effect on the mutant one (Figure 5C,D). To test whether they interacted with each other, we conducted RNA pull‐down assay in Hep‐2 and TU212 cells. As shown in Figure 5E, compared with control probe, circ‐CCND1 probe could abundantly pulled down miR‐466. Likewise, circ‐CCND1 and CCND1 3′‐UTR were substantially enriched by wild‐type miR‐646 probe, but not by the mutated probe (Figure 5F). Depletion of circ‐CCND1 significantly up‐regulated miR‐646 expression (Figure 5G). Besides, the diminished CCND1 mRNA and proliferative abilities of Hep‐2 and TU212 cells caused by circ‐CCND1 silencing were partly rescued by miR‐646 silencing and were wholly rescued after miR‐646 knockdown combined with HuR overexpression (Figure 5H, I). In all, these above results demonstrate that circ‐CCND1 can stabilize the mRNA of its host gene via HuR and miR‐646 in LSCC (Figure 5J).

## 4 DISCUSSION

Emerging evidence suggests that there is a cross‐link between circRNA and its parental gene. circRNA can regulate its parental gene expression in a *cis‐* or *trans‐*acting manner. In this study, we characterized a CCND1‐derived circRNA, referred to as circ‐CCND1, which positively regulated CCND1 expression via affecting its stability. circ‐CCND1 was notably overexpressed in LSCC and closely associated with malignant clinical behaviours and dismal outcome. Functional experiments showed that circ‐CCND1 was a positive regulator for LSCC cell proliferation. Mechanistically, cytoplasmic localized circ‐CCND1 was capable of directly interacting with HuR and miR‐646 to coordinately enhance CCND1 mRNA stability, leading to increased CCND1 expression, thereby facilitating LSCC growth. Therefore, our data highlight the biological relevance of circRNA in LSCC and also advance the understanding of the regulatory role of circRNA on its host gene.

circRNA is characterized by the covalently closed loop structure without 5′‐end cup and 3′‐end ploy‐A tail. It is widely expressed in eukaryotic cells and possesses important gene regulatory potential, which makes it involved in tumorigenesis and aggressiveness.^15^ Recent studies have shown that circRNA functions through different actions, including serving as ‘miRNA sponge’, regulation of transcription and splicing, interacting with RNA‐binding protein, and even translating polypeptide.^16^ The most extensively studied is circRNA as a competing endogenous RNA (ceRNA) to effectively sponge miRNA, for example, circ‐HIPK3/miR‐7 in colorectal cancer,^17^ circ‐EPSTI1/miR‐942 in ovarian cancer,^18^ circ‐ANKS1B/miR‐148/152 in breast cancer^19^ and circ‐AMOTL1L/miR‐193a‐5p in prostate cancer.^20^ It is well recognized that miRNA is a post‐transcriptional regulator by decreasing mRNA stability or inhibiting translation via directly targeting gene 3′‐UTR.^21^ In the current study, we identified that circ‐CCND1 could simultaneously sponge three miR‐646 to diminish the repression of miR‐646 on CCND1 3′‐UTR, resulting in enhancing the stability of CCND1 mRNA. Growing evidence indicates that miR‐646 plays a tumour‐suppressive role in a variety of human cancers, including osteosarcoma,^22^ hepatocellular carcinoma^23^ and gastric cancer.^24^ Likewise, we found that miR‐646 was remarkably up‐regulated after oncogenic circ‐CCND1 depletion, and knockdown of miR‐646 significantly rescued the decreased proliferation rate induced by circ‐CCND1 silencing, implying that miR‐646 may be also an anti‐cancer gene in LSCC. Therefore, these data suggest that circ‐CCND1 partially regulates the stability of its host gene via functioning as a ceRNA in LSCC.

On the other hand, circRNA is capable of physically interacting with RNA‐binding protein to regulate gene expression.^25^ Herein, we found that circ‐CCND1 could directly bind to HuR to potentiate CCND1 mRNA stability. HuR is a well‐known RNA‐binding protein that prevents mRNA decay via targeting the uridylate‐rich element (ARE, (A/U)UUU(A/U)) on the 3′‐UTR.^26^ A wealth of studies have demonstrated that HuR is ubiquitously expressed in eukaryotic organization and frequently elevated in human cancers, including LSCC.^27^ CCND1 is one of the best‐known HuR targets, in which HuR increases the half‐life of CCND1 by the ARE motif on CCND1 3′‐UTR.^28^ In our study, circ‐CCND1 was shown to interact with HuR and facilitate its enrichment on CCND1 3′‐UTR, thereby increasing CCND1 mRNA stability. Through sequence analysis, we found that there are two ARE motifs on the 3′‐UTR of CCND1 mRNA, which do not overlap with the binding site of miR‐646 (GCUGCU). Although some evidence shows that the interaction of HuR with the 3′‐UTR alters the conformation of mRNA, thereby competitively blocking the binding of miRNA to mRNA 3′‐UTR,^29^ our data displayed that exogenous HuR expression did not affect the interaction between miR‐646 and CCND1 mRNA 3′‐UTR (data not shown), suggesting that CCND1 mRNA stability regulated by circ‐CCND1 was independently controlled by HuR and miR‐646 in LSCC. Whether circ‐CCND1 also plays a similar regulatory role in other malignant tumours warrants further investigation.

Collectively, our findings clearly suggest that circ‐CCND1 functions as a novel positive regulator for LSCC growth through post‐transcriptional alteration of CCND1 stability via coordinated regulation of HuR and miR‐646, and also lay a foundation for the pursuit of circ‐CCND1 as a promising prognostic biomarker and druggable target for LSCC patients.

## CONFLICT OF INTEREST

The authors declare no conflict of interest.

## AUTHOR CONTRIBUTIONS

BLW designed the study. YZZ, JL and YT performed the experiments and analysed the data. YZZ and BLW wrote and revised the manuscript.

## Data Availability

The data sets and supporting materials generated and/or analysed during the current study are available from the corresponding author on reasonable request.
